# RhoA/Rho-kinase triggers epithelial-mesenchymal transition in mesothelial cells and contributes to the pathogenesis of dialysis-related peritoneal fibrosis

**DOI:** 10.18632/oncotarget.24208

**Published:** 2018-01-12

**Authors:** Qinglian Wang, Xiaowei Yang, Ying Xu, Zhenwei Shen, Hongxia Cheng, Fajuan Cheng, Xiang Liu, Rong Wang

**Affiliations:** ^1^ Department of Nephrology, Shandong Provincial Hospital Affiliated to Shandong University, Jinan, China; ^2^ Department of Biostatistics, School of Public Health, Shandong University, Jinan, China; ^3^ Department of Pathology, Shandong Provincial Hospital Affiliated to Shandong University, Jinan, China

**Keywords:** peritoneal dialysis, peritoneal fibrosis, EMT, RhoA/Rho-kinase, AGEs

## Abstract

Peritoneal fibrosis (PF) with associated peritoneal dysfunction is almost invariably observed in long-term peritoneal dialysis (PD) patients. Advanced glycation end products (AGEs) are pro-oxidant compounds produced in excess during the metabolism of glucose and are present in high levels in standard PD solutions. The GTPase RhoA has been implicated in PF, but its specific role remains poorly understood. Here, we studied the effects of RhoA/Rho-kinase signaling in AGEs-induced epithelial-mesenchymal transition (EMT) in human peritoneal mesothelial cells (HPMCs), and evaluated morphological and molecular changes in a rat model of PD-related PF. Activation of RhoA/Rho-kinase and activating protein-1 (AP-1) was assessed in HPMCs using pull-down and electrophoretic mobility shift assays, respectively, while expression of transforming growth factor-β, fibronectin, α-smooth muscle actin, vimentin, N-cadherin, and E-cadherin expression was assessed using immunohistochemistry and western blot. AGEs exposure activated Rho/Rho-kinase in HPMCs and upregulated EMT-related genes via AP-1. These changes were prevented by the Rho-kinase inhibitors fasudil and Y-27632, and by the AP-1 inhibitor curcumin. Importantly, fasudil normalized histopathological and molecular alterations and preserved peritoneal function in rats. These data support the therapeutic potential of Rho-kinase inhibitors in PD-related PF.

## INTRODUCTION

Peritoneal dialysis (PD), a life-saving renal replacement strategy for patients with end-stage renal disease, is based on the transcapillary ultrafiltration capacity of the peritoneum, a semi-permeable membrane across which the exchange of waste products and solutes takes place [1, 2]. Compared to hemodialysis, house-based PD provides better preservation of residual renal function and improves quality of life. Nevertheless, continuous exposure to bio-incompatible dialysis solutions often causes repeated episodes of peritonitis, which together with glucose degradation products (GDPs) formed during heat sterilization of the solutions, and advanced glycation end-products (AGEs) produced in the peritoneum from glucose metabolism, cause inflammation and injury to the peritoneal membrane and result in progressive peritoneal fibrosis (PF) and tissue angiogenesis [3–5]. These morphological alterations are associated with ultrafiltration failure and increased rates of small-solute transport [3, 6]. PD solutions include highly concentrated glucose, an acidic pH, and GDPs, and are thus believed to be the major causative factor of PF in dialyzed patients [7–11]. However, the pathophysiologic mechanisms of PD-related PF are not completely understood. It has been demonstrated that, soon after PD is initiated, peritoneal mesothelial cells (PMCs) show a progressive loss of epithelial phenotype and acquire myofibroblast-like characteristics consistent with epithelial-mesenchymal transition (EMT) [12–15]. Since EMT is associated with pro-fibrotic signaling, it has been ascribed an important role in the development and progression of PF.

Research has shown that the high concentrations of glucose and GDPs in standard PD fluids induce a local diabetic environment, leading to the formation of AGEs and induction of EMT in PMCs [16, 17]. Moreover, several studies reported the presence of AGEs in PD patients’ peritoneal effluent and tissue, and outlined the clinical factors associated with their contribution to PF [11, 18, 19].

The Rho family of small GTPase proteins control a wide variety of cellular processes. RhoA is one of the best-known members of this family and the Rho kinases are the first and the best-characterized RhoA effectors. Among critical cellular processes regulated by RhoA/Rho-kinase signaling are contraction, motility, proliferation, differentiation, and apoptosis [20]. Rho-kinase inhibitors have demonstrated anti-fibrotic effects in several experimental models, including diabetic and non-diabetic chronic kidney diseases, among them glomerulosclerosis [21–23] and tubulointerstitial fibrosis [24]. The RhoA/Rho-kinase pathway is activated in the kidney by the diabetic milieu, and this contributes to abnormal cell growth and pro-sclerotic signaling with enhanced extracellular matrix (ECM) production [25, 26]. Accordingly, the use of Rho-kinase inhibitors such as Y-27632 and fasudil (HA-1077) yielded promising results in animal models of renal and peritoneal fibrosis. For example, Y-27632 was shown to prevent tubulointerstitial fibrosis in the mouse kidney with unilateral ureteral obstruction [24], while fasudil ameliorated some of the morphological and functional alterations found in rat models of encapsulating peritoneal sclerosis and dialysis-related PF [27, 28]. However, the mechanisms underlying these protective effects have not been explored in depth.

RhoA regulates the formation of F-actin stress fibers and focal adhesion complexes [20]. Through activation of serum response factor, RhoA affects the transcription of genes containing the serum response element [29]. Among these, the most well-characterized are c-fos and c-jun, components of the transcription factor activating protein 1 (AP-1). RhoA activates AP-1 in multiple cells, including renal mesangial cells and fibroblasts, where it regulates numerous genes involved in the fibrotic response [26, 30–32]. These include some genes characteristically upregulated in diabetic nephropathy such as TGF-β, plasminogen activator inhibitor-1, and fibronectin (FN) [32–34]. It is unclear, however, whether RhoA-mediated AP-1 transcriptional activity influences EMT in PMCs and contributes to PF in dialysis patients.

The present study aimed to test the hypothesis that AGEs activate RhoA/Rho-kinase signaling and induce EMT in PMCs, and to define the role of this signaling pathway in an animal model of PD-related PF. Our results suggest that RhoA/Rho-kinase pathway activation induces EMT in AGEs-exposed PMCs, and is a major factor in the development of PD-related PF.

## RESULTS

### AGEs exposure activates the RhoA/Rho-kinase pathway in HPMCs

**Figure 1:**
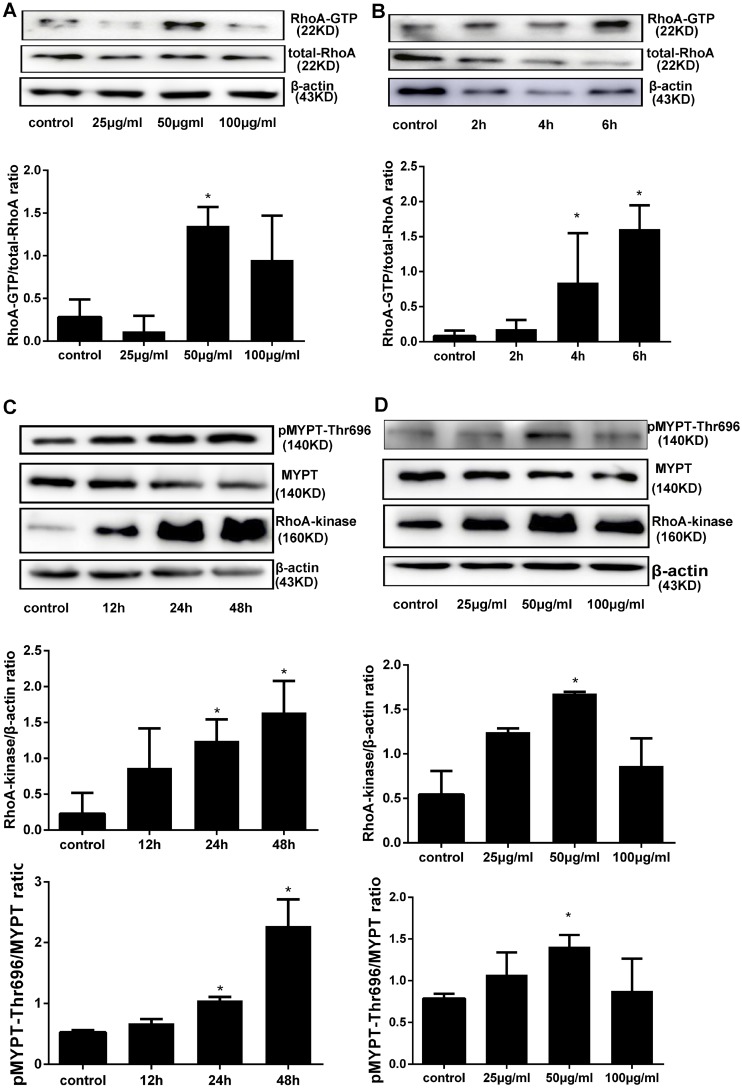
AGEs activate the RhoA/Rho-Kinase pathway in HPMCs (**A**) RhoA pull-down activity assay in HPMCs treated for 6h with increasing concentrations of AGEs. (**B**) Time course of RhoA activation in cells treated with 50 μg/ml AGEs. (**C**) Assessment of total Rho-Kinase protein and Rho-Kinase activation (by detection of MYPT1 phosphorylation at Thr696) after treatment with 50 μg/ml AGEs. D) Detection of Rho-Kinase and p-MYPT1-Thr696 after exposure to increasing concentrations of AGEs for 48 h. (Data are mean ± SD; ^*^P < 0.05 vs control, *n* = 3).

Increased RhoA activation was reported in different cell types after high glucose exposure. To assess if RhoA activation is associated with AGEs exposure in HPMCs, a RhoA pull-down activation assay was performed after short-term (6 h) treatment with different concentrations of AGEs. Maximal RhoA activity was observed after treatment with 50 μg/ml AGEs (Figure 1A). Subsequent time course experiments using this concentration showed a time-dependent increase in RhoA activation (Figure 1B). Next, downstream Rho-kinase activation was measured by assessing Thr696 phosphorylation on MYPT1 (p-MYPT1-Thr696), a specific Rho-kinase target, using western blot. As shown in Figure 1C–1D, increases in both RhoA-kinase expression and p-MYPT1-Thr696 were seen after AGEs-induced RhoA activation.

### RhoA/Rho-kinase pathway inhibition prevents AGEs-induced EMT

**Figure 2:**
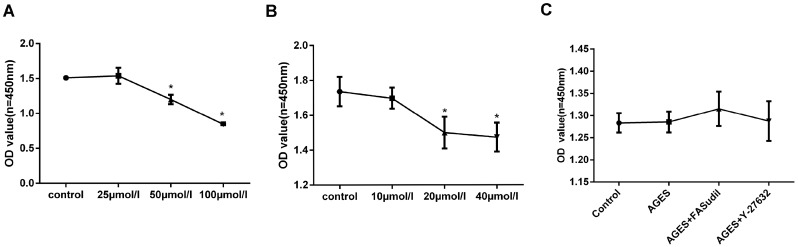
Cell viability assays (**A**–**B**) Dose-dependent increase in cytotoxicity induced by fasudil and Y-27632 (48h incubation). (**C**) Treatment with AGEs (50 μg/ml), fasudil (25 μmol/l), or Y-27632 (10 μmol/l) for 48h induced no obvious cytotoxicity. (Data are mean ± SD; ^*^P < 0.05 vs control, *n* = 3).

Stimulating AGEs concentrations and times were chosen based on results of preliminary experiments, and the Rho-kinase inhibitors fasudil and Y-27632 were used according to our own preliminary data and previous studies [26]. First, we evaluated the potential cytotoxic effects of AGEs, fasudil, and Y-27632 on HPMCs. No significant toxicity was detected on WTS-based viability assays for 25 μmol/l of fasudil, 10 μmol/l of Y-27632, and 50 μg/ml of AGEs (Figure 2), and these concentrations were chosen for subsequent experiments.

**Figure 3:**
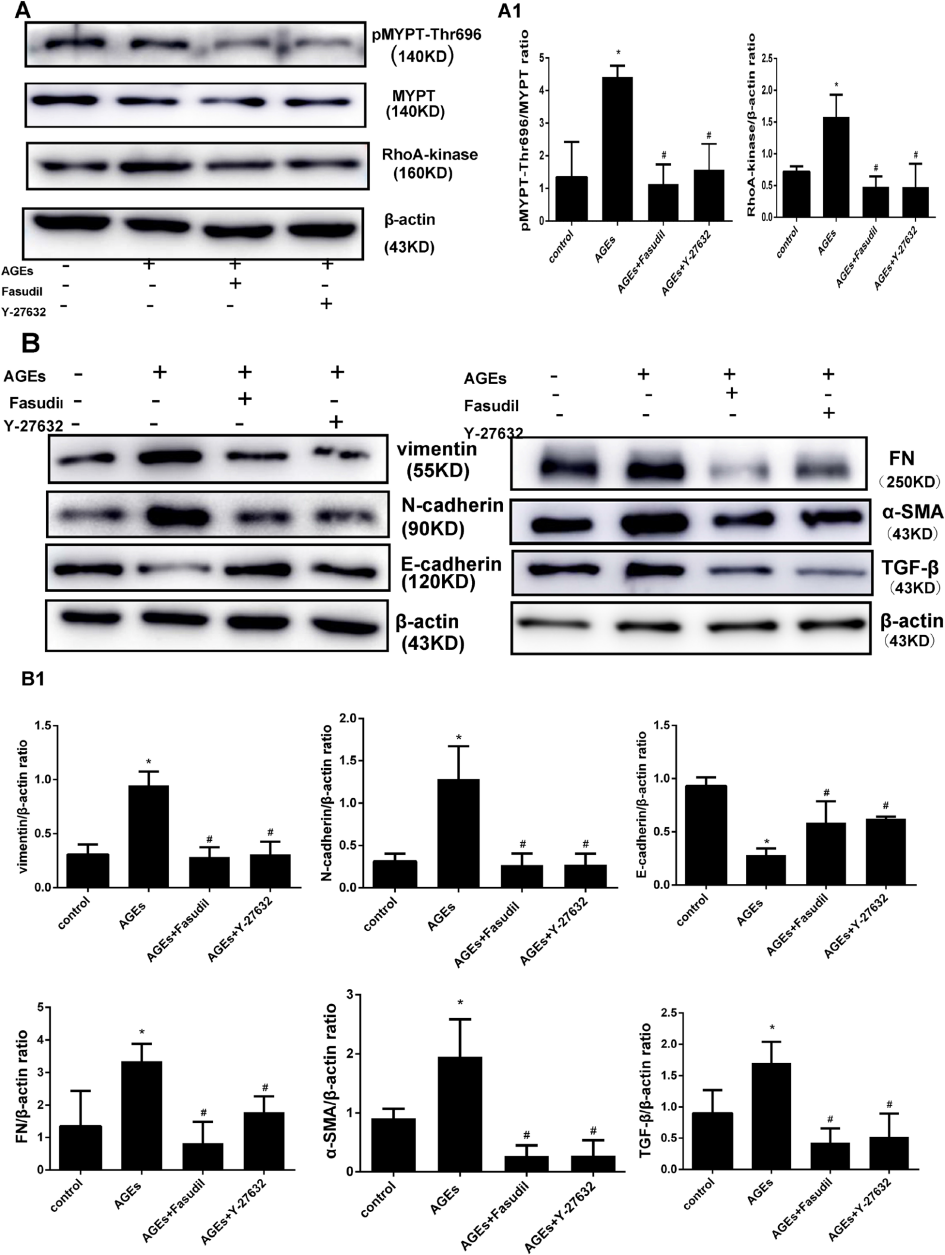
Fasudil and Y-27632 inhibit AGEs-induced RhoA signaling and EMT (**A**) Western blot detection of Rho-Kinase and p-MYPT-Thr696 in HPMCs treated with fasudil (25 μmol/l) or Y-27632 (10 μmol/l) 30 min before exposure to AGEs (50 μg/ml). A1) Quantitative analysis of data shown in A. (**B**) Western blot detection of vimentin, N-cadherin, E-cadherin, FN, α-SMA, and TGF-β. B1) Quantitative analysis of data shown in B. (Data are mean ± SD; ^*^P < 0.05 vs control, ^#^*P* < 0.05 vs AGEs, *n* = 3).

EMT markers such as TGF-β, FN, α-SMA, vimentin, and N-cadherin are upregulated while E-cadherin is downregulated during EMT in MCs. To determine whether expression of these proteins is dependent on RhoA/Rho-kinase pathway activation, HPMCs were exposed to AGEs and Rho-kinase inhibitors. Pre-treatment (30 min) with fasudil and Y-27632 markedly inhibited the increases in Rho-kinase levels and p-MYPT1-Thr696 elicited by AGEs (Figure 3A). On the other hand, AGEs exposure increased the expression of TGF-β, FN, α-SMA, vimentin, and N-cadherin, and decreased the expression of E-cadherin, and these changes were prevented by both fasudil and Y-27632 (Figure 3B–3C).

**Figure 4:**
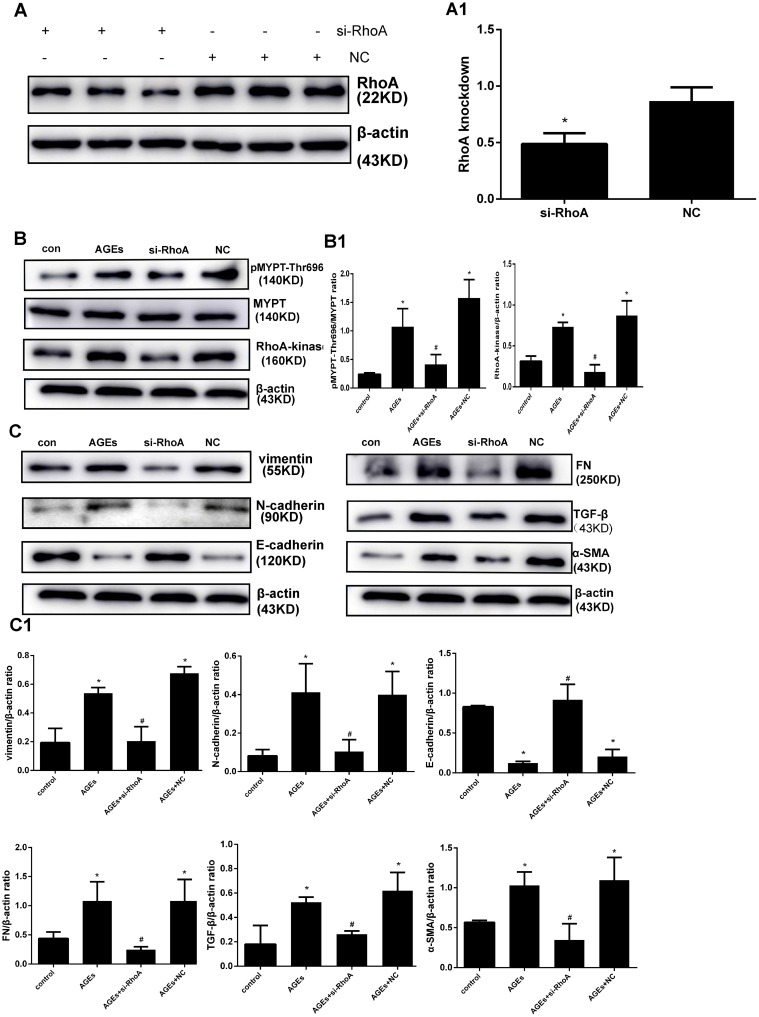
RhoA silencing abrogates AGEs-induced EMT (**A**) Knockdown efficiency of si-RhoA in HPMCs. A1) Semiquantitative analysis of si-RhoA silencing efficiency. (**B**) Downregulation of Rho-Kinase and p-MYPT-Thr696 after RhoA silencing. B1) Semiquantitative analysis of data shown in B. (**C**) Reversal of AGEs-induced upregulation of EMT markers by RhoA knockdown; non-transfected or NC-transfected HPMCs served as controls. C1) Semiquantitative analysis of data shown in C. (Data are mean ± SD; ^*^P < 0.05 vs control, ^#^*P* < 0.05 vs AGEs, *n* = 3).

Subsequently, the effects of RhoA knockdown were assessed in AGEs-treated HPMCs transfected with RhoA-specific siRNA. Successful inhibition of RhoA expression (Figure 4A) correlated with downregulation of p-MYPT1-Thr696, Rho-kinase (Figure 4B) and EMT markers (Figure 4C) were showed after exposure to AGEs. These results suggest that RhoA/Rho-kinase pathway activation mediates AGEs-induced EMT in HPMCs.

### RhoA/Rho-kinase pathway activation mediates AGEs-induced HPMC migration

**Figure 5:**
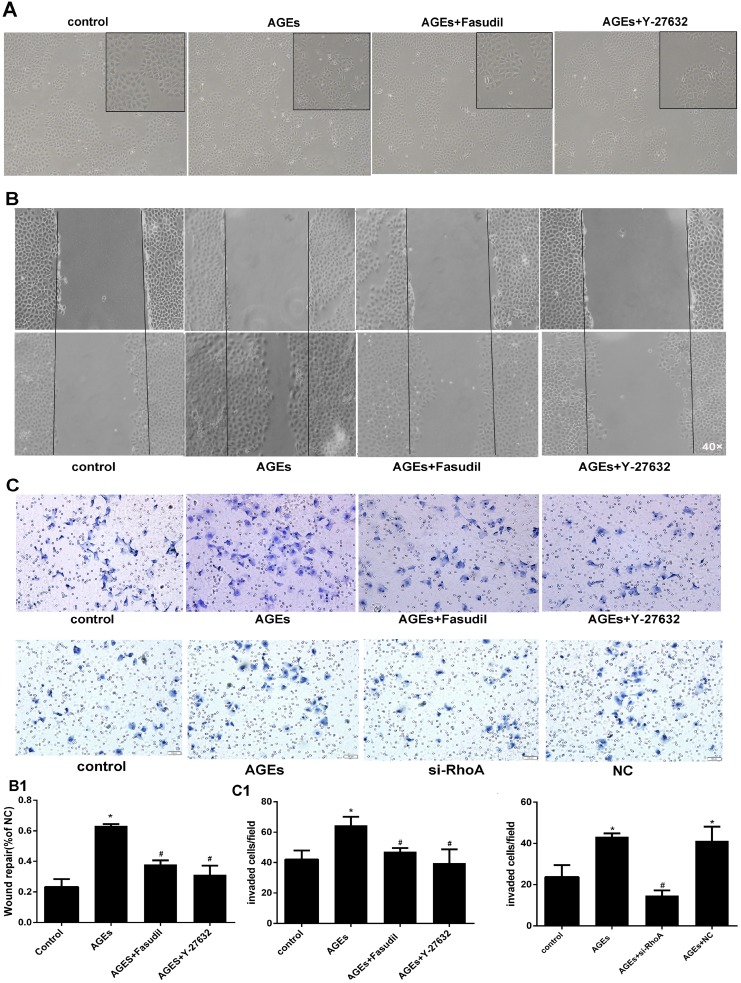
RhoA inhibition decreases AGEs-induced migration in HPMCs (**A**) Morphological changes in HPMCs. After 48h incubation with AGEs, the typical cobblestone-like growth pattern disappears and cells became thin and slender. Fasudil and Y-27632 prevented these changes. (**B**) Coordinated sheet migration in cells treated with AGEs in the presence or absence of fasudil and Y-27632 (wound-healing assay). B1) Quantitative analysis of wound closure. (**C**) Transwell migration assay results in HPMCs treated with AGEs with or without pre-treatment with Rho-kinase inhibitors or si-RhoA. Non-treated HPMCs served as controls. C1) Quantitative analysis migration in the transwell assay. (Data are mean ± SD, ^*^P < 0.05 vs control, ^#^*P* < 0.05 vs AGEs, *n* = 3).

Cultured HPMCs show a cobblestone appearance. Consistent with EMT development, after incubation with AGEs for 48h the cells become thin, slender, and resemble fibroblasts. In line with the preceding experiments, both fasudil and Y-27632 prevented these morphological changes (Figure 5A).

As cells undergoing EMT acquire higher mobility, wound healing and transwell migration assays were conducted to assess the influence of RhoA/Rho-kinase on AGEs-induced motility in HPMCs. Compared with BSA-treated (control) cells, AGEs exposure markedly increased cell migration, and this effect was reversed by both fasudil and Y-27632 in both the wound-healing (Figure 5B) and transwell (Figure 5C) assays. Taken together, these findings demonstrate that RhoA/Rho-kinase pathway activation mediates the increase in motility associated with EMT in AGEs-treated HPMCs.

### Rho-kinase signaling mediates AGEs-induced AP-1 activation in HPMCs

**Figure 6:**
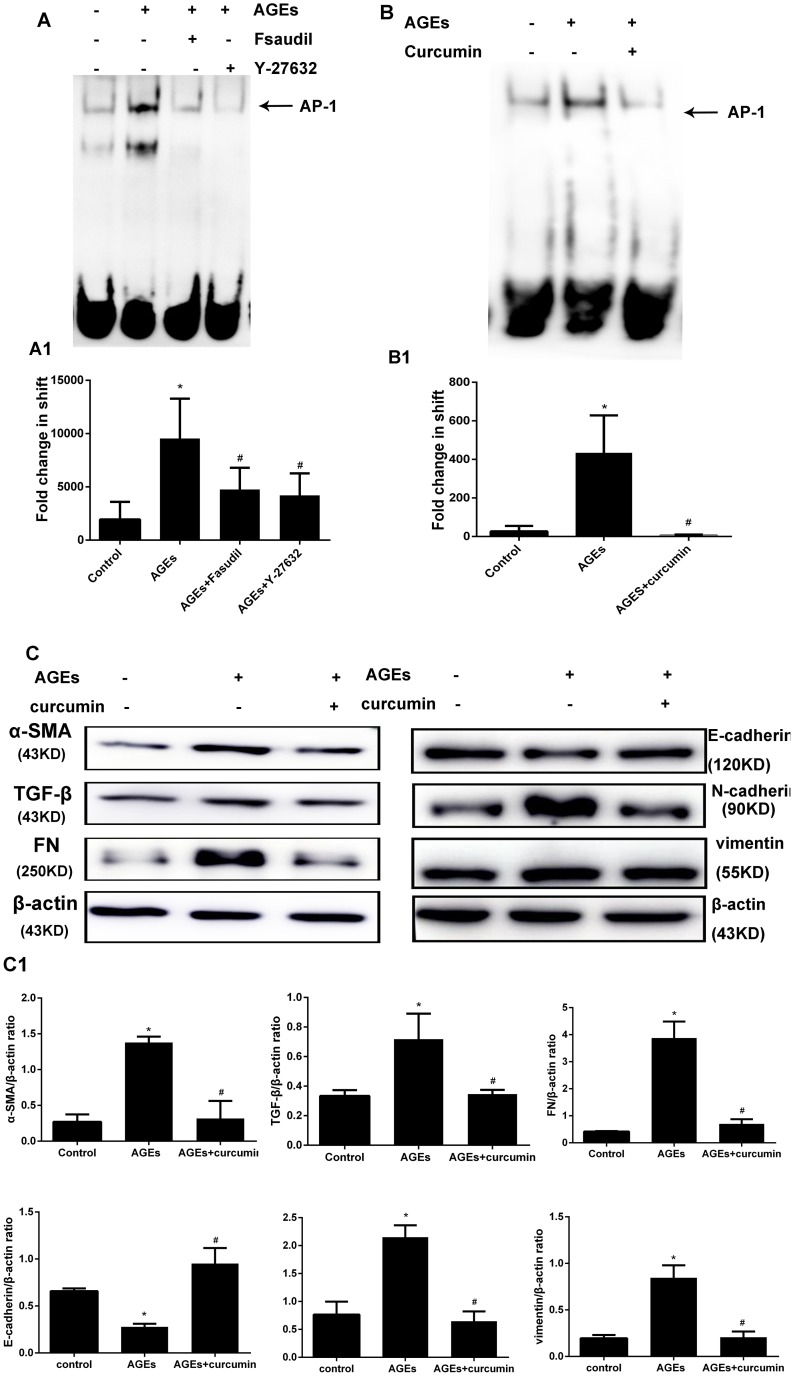
Rho-kinase mediates AGEs-induced activation of AP-1 in HPMCs (**A**) Detection of AP-1 activity by EMSA. HPMCs were pretreated with fasudil or Y-27632 before AGEs exposure. The assay was performed in nuclear protein extracts. A1) Semiquantitative analysis of data shown in A. (**B**) Inhibition of AP-1 activation by curcumin (20 μmol/l, 30 min treatment). B1) Semiquantitative analysis of data shown in B. (**C**) Western blot assessment of EMT markers in HPMCs incubated with AGEs (48h) in the presence or absence of curcumin. C1) Semiquantitative analysis of data shown in C (Data are mean ± SD, ^*^P < 0.05 vs control, ^#^*P* < 0.05 vs AGEs, *n* = 3).

To evaluate the mechanism by which AGEs-induced RhoA activation leads to increased expression of EMT markers, we investigated whether activation of the transcription factor AP-1 is induced by AGEs, and whether RhoA signaling is involved in this process. AP-1 transcriptional activity was evaluated by electrophoretic mobility shift assay (EMSA) using an AP-1 consensus oligonucleotide. As shown in Figure 6A, AGEs exposure increased AP-1 nuclear protein binding activity, and this effect was prevented by both fasudil and Y-27632. These results indicate that AGEs induce AP-1 activation through RhoA/Rho-kinase signaling.

### AP-1 inhibition prevents AGEs-induced EMT

To assess the influence of AP-1 on AGEs-induced EMT, the effects of the AP-1 inhibitor curcumin on AP-1 transcriptional activity and EMT marker expression were tested in HPMCs incubated with AGEs. Curcumin treatment effectively inhibited AP-1 activity (Figure 6B), and markedly reversed the changes in TGF-β, FN, α-SMA, vimentin, N-cadherin, and E-cadherin expression induced by AGEs exposure in HPMCs.

### Fasudil prevents histopathological changes and ultrafiltration deficits in rats with peritoneal fibrosis

**Figure 7:**
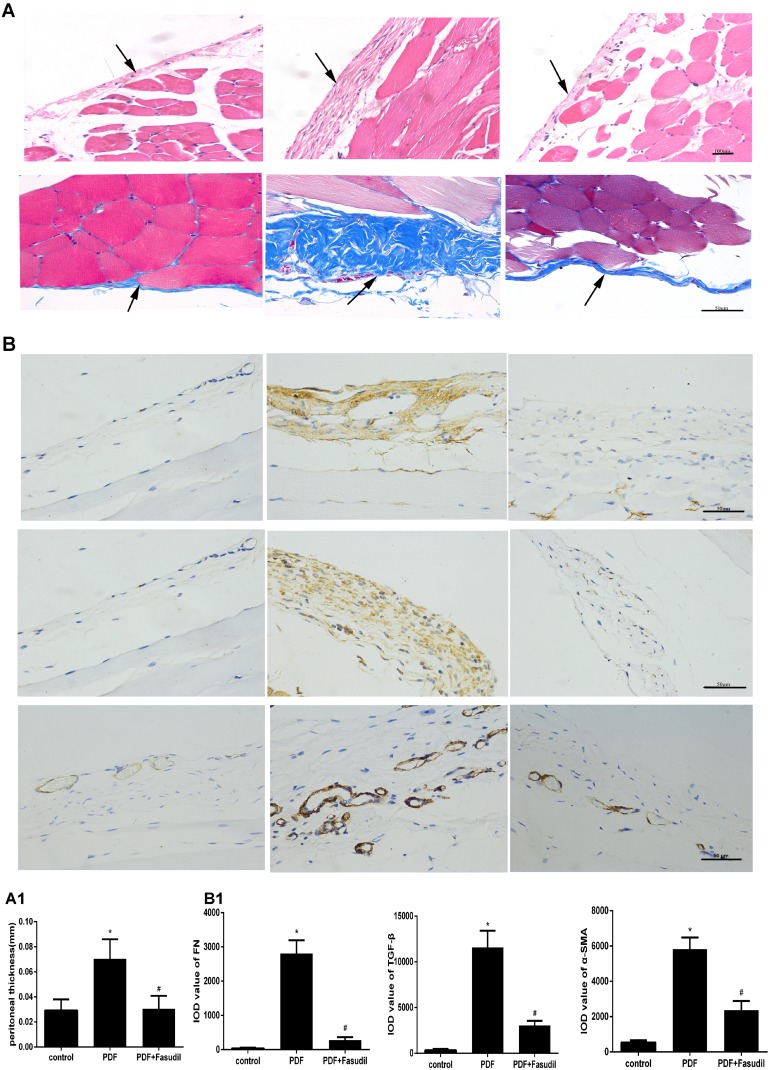
Fasudil reduces PD-associated peritoneal lesions in rats (**A**) H&E and Masson's trichrome staining of the peritoneum (H&E, 200×; Masson, 400×; bars: H&E, 100 μm; Masson, 50 μm). Note the markedly increased peritoneal thickness resulting from ECM deposition, and the significant attenuation of these pathological changes after fasudil treatment. (**A1**) Quantitative analysis of peritoneal thickness. (**B**) Immunohistochemical detection of TGF-β, FN, and a-SMA (400×; bar: 50 μm). (**B1**) Semiquantitative analysis of data shown in B. (Data are mean ± SD, ^*^P < 0.05 vs control, ^#^*P* < 0.05 vs AGEs, *n* = 3).

An animal model of PD-related PF was established by i.p. administration of high glucose-based peritoneal dialysis fluid (PDF) plus LPS. After sacrifice, H&E and Masson's trichrome staining were performed in sections of the peritoneum of PDF, PDF plus fasudil, and control (saline-injected) rats. Rats in the PDF-only group showed loss of the mesothelial cell monolayer, accumulation of inflammatory cells, and proliferation of fibroblasts (Figure 7A). In addition, compared with control animals, peritoneal thickness was markedly increased in these rats (70.5 ± 15.5 μm vs. 29.3 ± 8.7 μm). Notably, treatment with fasudil completely prevented peritoneal thickening (30.6 ± 10.3 μm, Figure 7A, Table 1).

**Table 1:** Body weight, ultrafiltration volume (UV), and peritoneal thickness

Group	Body weight (g)	UV (ml)	Peritoneal thickness (μm)
Control	285.25 ± 6.65	9.50 ± 2.52	29.2 ± 8.7
PDF	291.00 ± 11.64	2.39 ± 1.76^*^	70.5 ± 15.5^*^
Fasudil	285.38 ± 21.72	7.24 ± 1.36^#^	30.6 ± 10.3^#^

Data express mean ± SD. ^*^P < 0.05 vs control, ^#^*P* < 0.05 vs PDF group, *n =* 8/group.

The ultrafiltration volume is indicative of the functional status of the peritoneum. A significant decrease in peritoneal filtrate, consistent with severe peritoneal dysfunction, was observed in PDF animals (2.39 ± 1.76 ml versus 9.50 ± 2.52 ml in the control group). In contrast, peritoneal function was much improved in PDF rats treated with fasudil (7.24 ± 1.36 ml) (Table 1). None of these treatments affected the weights of rats (Table 1).

### Fasudil decreases the expression of TGF-β, FN, and α-SMA in the peritoneum of rats with peritoneal fibrosis

**Figure 8:**
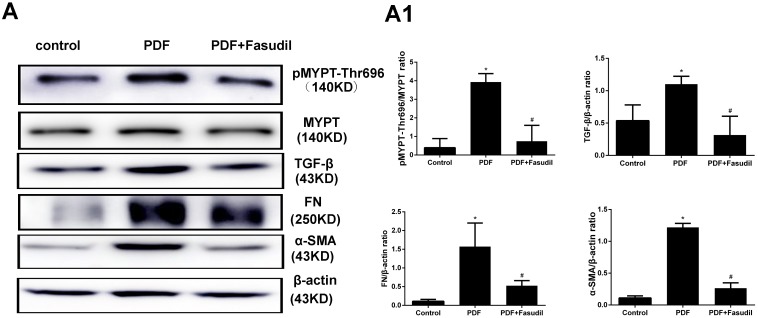
Fasudil prevents activation of Rho-kinase and reduces peritoneal fibrosis marker expression in rats **(A**) Western blot detection of p-MYPT-Thr696 and fibrosis-related factors in the visceral peritoneum. Note the marked downregulation of p-MYPT-Thr696, FN, TGF-ß, and α-SMA in samples from fasudil-treated rats. **(A1**) Semiquantitative analysis of data shown in A. (Data are mean ± SD, ^*^P < 0.05 vs control, ^#^*P* < 0.05 vs AGEs, *n* = 3).

To evaluate if the inhibitory effects of fasudil on AGEs-induced expression of EMT markers in cultured cells are reflected *in vivo*, TGF-β, FN, and α-SMA expression was assessed by immunohistochemistry and western blot in the peritoneum of rats with PD-related PF (Figure 7B). In line with the results obtained *in vitro*, TGF-β, FN, and α-SMA protein levels were significantly increased in PDF rats, and this changes were significantly attenuated by treatment with fasudil (Figure 8A).

## DISCUSSION

Long-term exposure of the peritoneum to hypertonic glucose-containing dialysis solution induces inflammation and a local diabetic environment associated with generation of AGEs and structural and functional deterioration of the peritoneal membrane [11, 18, 19]. In light of this, PD has been deemed analogous to “diabetes of the peritoneal cavity” [16]. Rho-kinase inhibitors have both anti-diabetic and anti-fibrotic properties, and their use provided beneficial effects in several experimental and clinical settings. However, the potential of Rho-kinase inhibitors as therapeutic agents to ameliorate peritoneal membrane damage resulting from long-term dialysis, as well as their mechanisms of action, have not yet been explored in depth [27, 28, 35].

In this study, we used *in vitro* and *in vivo* strategies to model the pathological changes induced in the peritoneum by prolonged exposure, through dialysis, to supraphysiological glucose concentrations with subsequent formation of prooxidant AGEs. We used a direct assay of RhoA activity, which isolates the active GTP-bound molecule, to show that AGEs activate RhoA and its main downstream effector, Rho-kinase, and trigger EMT in human PMCs; the ensuing morphological and functional changes severely compromise the barrier function of the peritoneal mesothelium.

Previous data suggested that PD induces EMT in MCs, contributing to the development and progression of PF [12–15]. In the course of PD PMCs upregulate transcription factors of the snail, ZEB, and Twist families, which control the molecular reprogramming of EMT [36]. The whole process can be induced by several growth factors, including TGF-β, which has been identified as a key mediator of mesothelial EMT both *in vitro* [37] and *in vivo* [38]. TGF-β initiates EMT by enhancing the synthesis of α-SMA and the secretion of extracellular matrix proteins such as collagens I, III, and IV, FN, and laminin [38–40]. Additional changes during EMT include downregulation of epithelial markers such as E-cadherin, claudins, and occludin, and upregulation of mesenchymal markers such as N-cadherin, snail, and vimentin [6]. In this study, we specifically analyzed the expression of TGF-β, FN, α-SMA, vimentin, N-cadherin, and E-cadherin, which are well-characterized EMT markers.

AGEs have also been shown to induce EMT in MCs [17]. Several studies demonstrated the presence of AGEs in peritoneal effluents and peritoneal tissue in PD patients, and the degree of AGEs accumulation is associated with the length and frequency of PD and with the extent of fibrosis and functional decline of the peritoneal membrane. Our *in vitro* studies showed that exposure to AGEs upregulated TGF-β, FN, α-SMA, vimentin, and N-cadherin, and downregulated E-cadherin in HPMCs, and these effects were abolished by both siRNA-mediated RhoA silencing and the Rho-kinase inhibitors fasudil and Y-27632. These data provide strong evidence that blocking the RhoA/Rho-kinase pathway prevents AGEs-induced EMT in HPMCs and suggest, in line with a study by Zhang et al. showing a role for RhoA/Rho-kinase signaling in TGF-β1-induced EMT in rat PMCs [35], the potential therapeutic value of RhoA-kinase inhibitors in the treatment of PD-related PF.

AP-1, one of the first mammalian transcription factors identified, regulates a wide range of cellular processes including proliferation, apoptosis, survival, and differentiation [41]. RhoA regulates the transcription of *c-fos* and *c-jun*, which are main AP-1 components [29]. Using EMSA, we confirmed AP-1 activation by AGEs in HPMCs, and demonstrated the involvement of RhoA/Rho-kinase signaling. The effect on AP-1 was functionally relevant, since RhoA/Rho-kinase inhibition prevented AGEs-induced upregulation of the AP-1 target genes TGF-β and FN [33, 34], as did AP-1 inhibition with curcumin. However, since curcumin also targets other transcription factors such as STAT and NF-κB [42], further experiments using specific AP-1 inhibitors are needed to validate these results and clarify whether α-SMA, vimentin, N-cadherin, and E-cadherin are also targets of AP-1.

Given these observations, we went on to assess the effects of fasudil, an orally bioavailable Rho-kinase inhibitor, in rats with PF induced by short-term LPS stimulation and chronic i.p. administration of a 4.25% dextrose-monohydrate dialysis solution (DIANEAL). We choose this PF model because continuous exposure to non-physiological PD solutions and episodes of peritonitis are presently the main cause of peritoneal dysfunction. After a 4-week model-building, the peritoneum showed expansion of the submesothelial compact zone, accumulation of extracellular matrix, loss of mesothelial cell layer integrity, and hypercellularity. Our *in vivo* results showed that fasudil administration notably improved peritoneal function and relieved peritoneal fibrosis by decreasing the thickness of the peritoneum, reducing extracellular matrix accumulation, and inhibiting the expression of TGF-β, FN, and α-SMA in the visceral peritoneum.

The therapeutic benefits of Rho-kinase inhibition may extend to other PF models. In a model induced by chlorhexidine [27], peritoneal damage including peritoneal thickening and fibrosis, as well as macrophage migration, and angiogenesis were ameliorated by fasudil treatment, as was the expression of TGF-β, FN, α-SMA, and vascular endothelial growth factor (VEGF). In human pleural mesothelial cells, high glucose increased TGF-β expression and VEGF secretion, which were blocked by Y-27632. In a second model induced by 4.25% DIANEAL PDF, fasudil significantly downregulated both mRNA and protein levels of TGF-β, α-SMA, and collagen in a dose-dependent manner, and reduced VEGF expression and peritoneal microvessel density [28]. These findings reinforce the therapeutic potential of Rho-kinase inhibitors for prevention and treatment of PF induced by long-term PD.

As EMT in MCs is central in the onset and progression of PF, substantial benefit is expected from therapeutic strategies designed to prevent or reverse EMT or its effects such as cellular invasion, ECM accumulation, and the synthesis of angiogenic factors [6]. Earlier studies have shown that antioxidants, ACEI/ARB, PPAR- γ agonists, and rapamycin were able to prevent PF through different mechanisms [43–46]. Nevertheless, these strategies are not fully effective. The RhoA/Rho-kinase pathway regulates numerous fundamental cellular functions, and its inhibitor fasudil has been used clinically in China and Japan. Fasudil has also shown benefits in animal models of different diseases involving tissue fibrosis. In light of all this evidence, further studies to clarify the cross-talk among Rho-kinase signaling and other pathways are warranted.

In conclusion, our studies reveal a critical role for RhoA/Rho-kinase signaling in AGEs-induced EMT in HPMCs, and during PD-related PF *in vivo*. The clinical use of Rho-kinase inhibitors represents a promising novel therapy to prevent or ameliorate PF in dialysis patients.

## MATERIALS AND METHODS

### Cell culture

The HPMC cell line (HMrSV5) was cultured in Dulbecco's Modified Eagle's Medium: Nutrient Mixture F-12 (DMEM-F12; HyClone, Carlsbad, CA, USA), supplemented with 15% fetal bovine serum (FBS; HyClone), streptomycin (100 μg/ml), and penicillin (100 units/ml) at 37°C in a 95% air/5% CO_2_ humidified atmosphere. AGEs (Beijing Biosynthesis Biotechnology, China), Y-27632 (Sigma, USA), Fasudil/HA-1077 (Sigma, USA), and curcumin (Sigma, USA) were prepared in DMSO to produce either 10 mM (AGEs) or 100 mM stocks and stored at 20°C.

Confluent cells were rendered quiescent by incubation for 24h in serum-free medium before being treated with AGEs (50 μg/ml) or BSA (50 μg/ml; osmotic control) for various times as indicated. The Rho-Kinase inhibitors Y-27632 (10 μmol/l) and fasudil /HA-1077 (25 μmol/l were added 30 min before addition of AGEs. The inhibitory activity and specificity of both inhibitors toward Rho-kinase have been demonstrated previously [47]. The AP-1 inhibitor curcumin (20 μmol/l) was also added 30 min before AGEs.

### RNA interference

A small interference RNA (siRNA) targeting RhoA (si-RhoA) and a negative control RNA probe (NC) were designed and purchased from Takara (Dalian, China). The corresponding sequences were as follows: 5′-UCAAGCAUUUCUGUCCCAA-3′ (si-RhoA) and 5′-AGUUCAACGACCAGUAGUC-3′ (NC). HPMCs were transfected using Lipofectamine 2000 reagent (Invitrogen, Carlsbad, CA, USA) according to the manufacturer's instructions. Western blot analysis was used to validate the efficiency of RhoA gene knockdown. After a 6h incubation with si-RhoA or NC, medium was changed to DMEM-F12 for another 24h. PMCs were then treated with or without AGEs (50 μg/ml) for an additional 48h. Non-transfected cells incubated in normal culture medium with BSA for 48 h served as controls.

### Cell proliferation assays

When cell density reached 70%, cells were seeded in 96-well plates at a density of 1,000 cells/well in 90 μl of complete medium supplemented with increasing concentrations of drugs. After 48h, 10 μl WST-8 solution (WST-8 cell proliferation and cytotoxicity assay kit; Dojindo, Kumamoto, Japan) was added into each well. After incubation for an additional 2h at 37°C, the absorbance was determined at 450 nm using a microplate reader (EL340 BioTek Instruments, Hopkinton, MA, USA). Untreated HPMCs were used as controls, against which all other results were normalized.

### Wound healing assays

Cells were seeded onto 6-well plates and allowed to grow to 90% confluence. A sterile 20 μl micropipette tip was used to scratch the cell monolayers. Then cells were washed with 1 ml PBS and supplemented with 2 ml serum-free DMEM-F12. Cells were incubated with AGEs and Rho inhibitors as described above. Pictures of migrating cells were taken at 0h and 12h. Migratory rates were calculated as (A-B)/A*100%, where A and B reflect the width of the wound at 0h and 12h respectively.

### Transwell migration assays

Transwell cell-culture inserts (pore size 8 μm; Corning Costar Corp., Cambridge, MA, USA) were placed in DMEM-F12 supplemented with 15% fetal bovine serum in the lower compartment. HPMCs with different pre-treatments were harvested with trypsin and re-suspended in serum-free DMEM-F12. The upper chambers were seeded with 1 × 10^5^ cells/ml, which were allowed to attach at 37°C for 12h. Then, non-migratory cells were removed from the upper surface of the chambers, and migrated cells in the lower membrane's surface were fixed with 4% paraformaldehyde and stained with hematoxylin. The number of migrated cells was counted in 3 separate fields per membrane at 200× using phase contrast microscopy (Leica Microsystems, GmbH). Data are presented as mean ± SD.

### RhoA pull-down assay

The RhoA pull-down assay was performed using the RhoA Pull-down Activation Assay Biochem Kit (BK036, Cytoskeleton, Inc, USA) following the protocol provided by the manufacturer. Briefly, cells were lysed in lysis buffer and supplemented with 1 mM Protease Inhibitor Cocktail. Cell lysates were harvested rapidly on ice with a cell scraper, and centrifuged at 15,000 rpm for 5 min at 4°C. At least 5 μl of lysate was saved for protein quantitation and 20–50 μg lysate was used for western blot. Equivalent lysate protein amounts (500 μg total cell protein) were added to a pre-determined amount of rhotekin-RBD beads at 4°C. Beads were incubated for 60 min with gentle rocking before collection by centrifugation at 15,000 rpm for 60s at 4°C. After being washed in washing buffer, beads were resuspended in 20 μl of 2× Laemmli sample buffer, then boiled for 2 min. The supernatant was resolved on 12% SDS-PAGE. Membranes were probed with anti-RhoA antibody (1 μg/ml; provided in the kit) and a species-specific horseradish peroxidase (HRP)-conjugated secondary antibody. Protein bands were imaged using an ECL system.

### Western blot analysis

Western blot was carried out as described previously [48]. Briefly, total protein was extracted from rat peritoneum or HPMCs in ice-cold lysis buffer containing 1 mmol/l proteinase and phosphatase inhibitors. Total protein (20 μg) was separated by 8–12% SDS-PAGE and transferred to polyvinylidene fluoride (PVDF) membranes. The membranes were then blocked with 5% non-fat dry milk for 1h at room temperature and incubated at 4°C overnight with primary antibodies raised against the following target proteins: rabbit anti-phospho-MYPT1 (Thr696) (1:1000; CST, Danvers, MA, USA), rabbit anti-MYPT1 (1:1000; CST), rabbit anti-RhoA-kinase (1:2000; Abcam, UK), rabbit anti-TGF-β (1:1000; Abcam), rabbit anti-α-SMA (1:1000; Abcam), rabbit anti-FN (1:1000; Proteintech, Rosemont, IL), mouse anti-E-cadherin (1:1000; Abcam), rabbit anti-N-cadherin (1:1000; Abcam), rabbit anti-vimentin (1:1000; Abcam), and rabbit anti-β-actin (1:1000; BOSTER, China). After incubation, membranes were washed three times with TBST for 10 min and re-probed with HRP-conjugated anti-mouse IgG, anti-rabbit IgG, or anti-goat IgG (1:5,000; Santa Cruz Biotechnology, Inc. Dallas, TX, USA) secondary antibodies for 1h at room temperature. Protein bands were detected using an ECL system and a Bio-Rad electrophoresis image analyzer (Bio-Rad, Hercules, CA, USA).

### Electrophoretic mobility shift assay

Nuclear protein extracts were prepared following manufacturer's instructions (P0028; Beyotime Biotechnology). Nuclear proteins (2 μg) were incubated for 20 min at room temperature with a biotin-labeled AP-1 consensus oligonucleotide (Sigma) (5›-CGC TTG ATG ACT CAG CCG GAA-3›, 3›-GCG AAC TAC TGA GTC GGC CTT-5›) as per the manufacturer's instructions (Thermo Fisher Scientific, Waltham, MA, USA). Reaction mixtures were electrophoresed in a 6% polyacrylamide gel, transferred, DNA cross-linked to a nylon membrane (Amersham; GE Healthcare, UK) and then probed with an HRP–conjugated streptavidin antibody (1:300). The membranes were washed with washing buffer for 5 min 3 times and incubated in equilibration buffer for 5 min before ECL detection.

### *In vivo* studies

The experimental protocols for all animal studies were approved by the Animal Ethics Committee of Shandong Provincial Hospital Affiliated to Shandong University. 8-week-old male Sprague-Dawley rats (130–140 g body weight, n = 24) were purchased from Shandong University Animal Center (Jinan, China). A rat peritoneal fibrosis model was generated by daily intraperitoneal (i.p.) injection of DIANEAL (4.25% dextrose-monohydrate peritoneal dialysis fluid; PDF, Baxter Healthcare Ltd., Deerfield, IL, USA) at 100 ml/kg for 4 weeks, combined with 4 initial injections of 0.6 mg/kg lipopolysaccharide (LPS; Santa Cruz, USA) on days 1, 3, 5, and 7 as described previously [49]. Rats were divided randomly into three groups (n = 8/group) and treated as follows: *Control*, 20 ml i.p. normal saline (NS) daily; *PDF*, 4.25% PDF (100 ml/kg) plus LPS i.p. as described above; *Fasudil*, same treatment as the *PDF* group plus gavage administration of the Rho-kinase inhibitor fasudil (30 mg/kg) daily for 3 weeks starting 24h after the last injection of LPS. Treatment was continued for 4 weeks, at which time rats were killed. Fasudil dosage was established according to previous reports [26].

Rats were weighed weekly. A peritoneal function test was performed on the last day of treatment. Rats were administered 25 ml of a 4.25% PDF solution before being anesthetized. Four hours later, the peritoneum was opened, and the net ultrafiltration volume, i.e. the volume of fluid recovered minus the volume of fluid administered, was measured. Both parietal and visceral peritoneum samples were harvested for regular histological evaluation and protein detection.

### Histopathological and immunohistochemical analyses

Parietal peritoneum tissues were routinely fixed in 10% neutral formalin and embedded in paraffin to obtain 3–4 μm thick serial tissue sections. Deparaffinized sections were stained with hematoxylin and eosin (H&E) and Masson's trichrome solution to analyze histopathological characteristics.

Before immunohistochemistry, tissue sections were heated using citrate buffer (pH 6.0) to unmask antigens. Samples were pre-treated with a 3% hydrogen peroxide solution to block endogenous peroxidase activity. Secondary goat anti-rat or anti-rabbit antibodies (Dako, Carpinteria, CA, USA) were applied as appropriate to detect primary anti-α-SMA (1:500, 13044; Google Biological Technology; Wuhan, China), anti-TGF-β (1:500, GB11034; Google Biological Technology), and anti-FN (1:500, 1574–1; Epitomics, USA) antibodies. Primary antibodies were incubated overnight at 4 °C in a humidity chamber, after blocking the slides for 30 min with 3% BSA. DAB (Dako) was used as HRP substrate for signal detection.

### Statistical analysis

Data are presented as mean ± SD unless stated otherwise. One-way ANOVA was used to determine statistical differences between groups. The Dunnett's test was used to perform multiple comparisons between groups. Two-tailed *P* < 0.05 indicated statistical significance. All statistical analyses were performed using SPSS 20.0 software (SPSS Inc. Chicago, Illinois, USA).

## Abbreviations

PF: peritoneal fibrosis
PD: peritoneal dialysis
PDF: peritoneal dialysis fluid
AGEs: advanced glycation end-products
GDPs: glucose degradation products
EMT: epithelial-mesenchymal transition
AP-1: activating protein-1
TGF-β: transforming grow factor-β
FN: fibronectin
α-SMA: α-smooth muscle actin
ECM: extracellular matrix
ACEI/ARB: angiotensin-converting enzyme inhibitor/angiotensin receptor blocker
PPAR-γ: Peroxisome proliferator-activated receptor gamma
PMCs: peritoneal mesothelial cells
HPMCs: human peritoneal mesothelial cells
